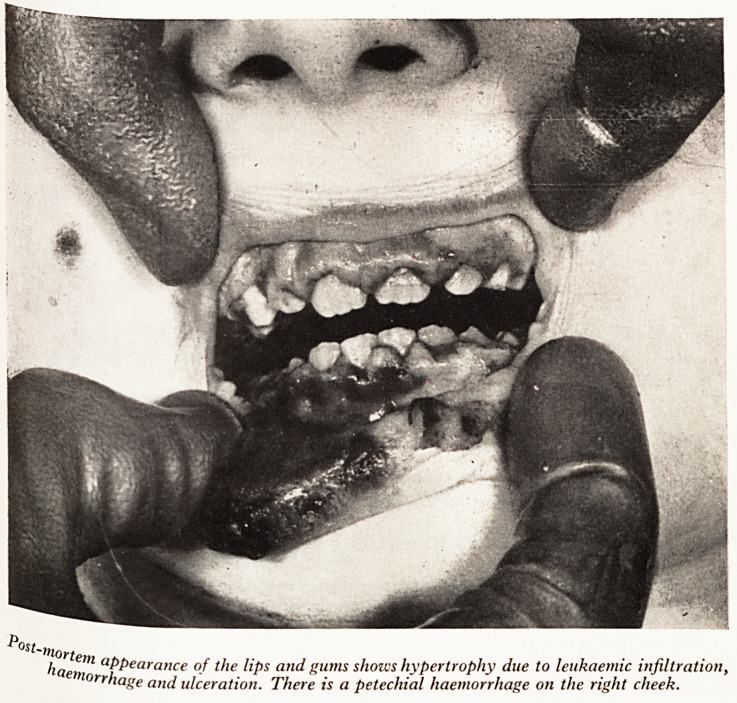# Astrocytoma Treated by Radiotherapy: Death from Acute Leukaemia

**Published:** 1957-04

**Authors:** T. F. Hewer


### ASTROCYTOMA TREATED BY RADIOTHERAPY: DEATH FROM ACUTE LEUKAEMIA

(*A Clinical Pathological Conference of the University of Bristol Medical School*)

CHAIRMAN: PROFESSOR T. F. HEWER

*Dr. Sluglett:* This is the case of a young girl whom I first saw in November 1952 at the age of six. She was brought in by her mother who said that she had been awake all night with earache. In the morning there was a discharge. She had an acute otitis with a perforated drum on the left side. She was treated with dry mopping and iodoform powder. She was subsequently referred to Mr. Scarff who, in April, removed her tonsils and adenoids. The next time I saw her was in July 1954. She was brought in by her mother who was worried about her squint which had developed recently. She was rather older than the age at which one normally sees squint but I could find no cause for it and she was otherwise perfectly well so I referred her to the Eye Hospital where she was seen by Mr. Brown and later admitted under Dr. Campbell in September.

*Mr. Hulme:* In the Eye Hospital she was seen by Dr. Campbell and later by Mr. Phillips and it was found that she had bilateral papilloedema and some impairment of vision in the left eye and bilateral external rectus weakness—she had a convergent squint. There was also slight weakness of her right leg. These findings suggested a cerebral tumour and she was transferred to Frenchay Hospital. Here a ventriculogram was performed (Plate III). Both lateral ventricles were found to be considerably dilated, the left being somewhat larger than the right, with deviation of the septum lucidum to the right. Only a little air entered the anterior part of the third ventricle and it was displaced towards the right. This suggested a space-occupying lesion related to the third ventricle and occluding it. This fitted the neurological findings of slight right-sided weakness. A tumour in this situation is likely to be inoperable. The next best thing is to circumvent the obstruction to the flow of cerebro-spinal fluid and treat the lesion with X-ray therapy, without necessarily performing a biopsy. No attempt was made to expose this tumour. A catheter was inserted into the left lateral ventricle and, passing it deep to the bone, was drained into the cisterna magna. This is an operation invented by a Norwegian, Torkildsen, and very valuable in the case of any lesion causing obstruction in the third ventricle or in the aqueduct. She then went to the General Hospital for X-ray therapy and did very well, remaining symptom-free although visual acuity on the left side was very considerably impaired. Her papilloedema subsided and at subsequent examination she showed neither motor weakness nor sensory loss and remained mentally perfectly normal. I saw her from time to time as an out-patient. Both her mother and I were very pleased with her progress. I last saw her on the 12th September, 1956 and she had been completely free of symptoms for the previous year although she now had a right homonymous visual field defect affecting mainly the temporal field of the right eye. It is possible that this may have been present for some time as in the young child it is very difficult to chart the visual fields accurately.

*Dr. Sluglett:* I saw her again on the 4th November, 1956 when her mother brought her in to my surgery and told me that she had started her periods. She was ten years old then and although this is an early age at which to start menstruation it is occasionally encountered and I suggested that if bleeding did not stop in a day or two we would go into the matter further. As they were about to go through the door her mother said: "By the way Doctor, I would like you to look at her gums and mouth. She is having trouble there." On looking in her mouth I noted the hyperlasia of the

1*

gums so I sent her to her dentist and, as we were both rather worried by the appearance we sent her to the Dental Hospital. I would emphasize that when I saw her she had no complaint. She appeared perfectly well.

*Dr. Apley:* My first acquaintance with this girl occurred when I was rung by the Dental Hospital and asked to see her because of an unusual condition of the gums. It had also been noted that there was a haemorrhagic rash on the body and that she had vaginal bleeding. I saw her in out-patients and arranged her immediate admission. It was apparent that the girl's condition was deteriorating with alarming rapidity. She was a big plump girl and, sure enough, she had signs of developing puberty with enlargement of the breasts and pubic and axillary hair. The vaginal bleeding was therefore not so out of the way as it might seem. She showed marked pallor, bleeding hypertrophied gums and some enlarged glands in the neck but not elsewhere and a petechial rash all over her body. The spleen was just palpable. On these findings we decided that this was almost certainly an acute leukaemia. She had the usual investigations done and these confirmed the diagnosis of leukaemia. The haemoglobin level was 60 per cent. Her white count was 140,000/c.m.m. and these cells were all atypical "blast" cells. There were no mature granulocytes.

Within a few hours her condition deteriorated quite markedly and by next day she was comatose. We gave her blood transfusions and also, on empirical grounds, we started her on cortisone. This drug does have a quite good effect in some cases, in others such as this it has no effect whatsoever. She became unresponsive, breathing was difficult and she could not swallow. She was developing signs of some intra-cranial lesion, her neck was stiff, her temperature was shooting up and she died within 48 hours of admission into hospital.

We were particularly interested in the diagnostic problem of how one could tie this acute leukaemia up with her history of tumour in the brain. Also there was the problem whether there was some connection with the irradiation that she had received.

*Prof. Perry:* Would a radiodiagnostician or neurosurgeon tell us what importance they attach to the very marked beaten silver appearance of the skull?

*Mr. Hulme:* Yes, she had evidence of increased intracranial pressure, the markings on the internal tables being due to pressure of the convolutions. There was no separation of the cranial sutures nor was there any evidence of erosion of the base although possibly the posterior clinoid processes are thinned.

*Dr. Cosh:* Have you a record of the C.S.F. pressure at that time?

*Mr. Hulme:* She had a lumbar puncture at the Eye Hospital and the pressure was then 300 mm. Hg but with small children this is not very reliable as they struggle and the pressure shoots up and down. It may have been 300 $\pm$100. One must remember that in order to get dilatation of ventricles of that extent there must have been obstruction for some considerable time.

*Prof. Hewer:* I know the neurosurgeons did not make any exploration of the tumour but have they any feelings as to what sort of a tumour it was?

*Mr. Hulme:* The most likely would be an infiltrating glioma. The tumour did appear to be to the side of the third ventricle rather than actually in it. It appeared to be arising in one of the basal ganglia and squashing rather than occluding the third ventricle.

*Dr. Cates:* I do not understand why you decided this was a tumour rather than a chronic abscess or tuberculoma.

*Mr. Hulme:* I was going on the grounds of probability. An abscess is most likely to be secondary to a middle ear infection and this would be in the temporal lobe which is not the case here.

*Dr. Apley:* Perhaps it is not realized how very common these intracranial tumours are in children. It is said that at least one-seventh of all intracranial tumours occur before the age of ten.

*Prof. Perry:* Is it not also true that, from Cushing's figures, quite a number of these space-occupying lesions were, as Dr. Cates suggests, inflammatory?

PLATE III

*Ventriculogram before by-pass operation shows dilatation of the lateral ventricles and failure of air to enter the third ventricle.*

ABOVE. *Sagittal section of brain at 70 mm. from frontal poles shows compression of the left globus pallidus and left optic tract by tumour in the wall of the third ventricle.*

BELOW. *Sagittal section at 80 mm. from frontal poles shows haemorrhages in the left thalamus and cerebral cortex.*

*Mr. Hulme:* Well, I do not think that is true of the cases which come to us at Frenchay.

*Prof. Hewer:* Probably they are very well selected before they come to you.

*Prof. Neale:* Did the papilloedema improve after the operation?

*Mr. Hulme:* Yes, it subsided completely. She did have some secondary optic atrophy.

*Mr. R. V. Cooke:* Does the apparently favourable effect of radiation prove or disprove any of these diagnoses? Is it probable that tumour was more likely in view of the apparently favourable effect of radiotherapy?

*Mr. Hulme:* The difficulty is that the effect of the relief of obstruction to the C.S.F. is difficult to assess. She might have improved equally without radiotherapy.

*Dr. Lloyd:* Was the irradiation applied only to the skull or to the spine as well?

*Mr. Hulme:* As far as I know only to the skull.

*Dr. Cates:* We now know that you were right and this was a tumour, but at the time had you really cleared up all the diagnostic difficulties before starting deep X-ray treatment?

*Mr. Hulme:* No, we hadn't. We have seen cases like this before and in trying to get at a tumour in this situation we could do an awful lot of damage. Even with a needle one may damage the basal ganglia.

*Prof. Hewer:* A needle biopsy would not have been a good thing if it were a tuberculoma either.

*Dr. Cates:* I don't know about that. You can cure it nowadays with streptomycin and I.N.H. It would be better than giving it radiotherapy.

*Dr. Apley:* Was a tuberculin test done?

*Mr. Hulme:* No, in fact it wasn't.

*Prof. Perry:* To go on to the leukaemia and the haemorrhage manifestations, I thought that in leukaemia the patient developed purpura because he ran out of platelets. Here is a patient developing colossal bleeding with a count of 50,000 platelets/c.mm. I asked Dr. Bolton about this and he said that he didn't think it could happen.

*Dr. Bolton:* This is, of course, a very low count.

*Prof. Neale:* What is the experimental error? Plus or minus 50,000! (Laughter.) To take a more clinical point, did this child's bilateral 6th nerve palsy improve as the result of treatment?

*Mr. Hulme:* I think it did. But of course her diplopia disappeared because of her visual accuity defect in the left eye. When I saw her in March 1955 she had no rectus palsy.

*Prof. Hewer:* Did the eye signs clear up immediately after the operation? If so, would you not think this was due to the reduction in pressure rather than the radiotherapy?

*Mr. Hulme:* Yes, it was due to the reduction in pressure. External rectus weakness may occur with any increase in intracranial pressure.

*Prof. Hewer:* I think we ought to have the post mortem now.

*Dr. Johnson:* The body was that of a moderately obese girl, fairly tall for her age and, as you have heard, she appeared to be approaching puberty. There was blood exuding from the nose and there was an extensive purpuric rash especially on the lower limbs and on the trunk. There was also a little oedema of the ankles. The brain was swollen with flattening of the cerebral convolutions and there was some blood beneath the pia at the vertex. The catheter inserted into the posterior part of the left lateral ventricle passed through the meninges and ran between the dura and the bone, backwards and down to empty into the cisterna magna. A solid whitish tumour 3·5 × 2·5 × 2·5 cm. extended upwards round the floor and left lateral wall of the third ventricle compressing the left globus pallidus, the left subthalamic nucleus and the left optic tract (Plate IV). An extension of the tumour (1·5 cm. diameter) in the region of the pituitary stalk pushed forwards the optic chiasma and had produced enlargement of the hypophyseal fossa and flattening of the pituitary gland. There was also a haemorrhage into the thalamus on the left side distinct from the brain tumour, occluding the lateral ventricle, and smaller haemorrhages into the cortex. The part of the brain adjacent to the tumour showed a moderate gliosis and also the presence of small calcified particles, calcospherites, which are frequently found in the neighbourhood of a slowly-growing brain tumour. The blood vessels in the tumour appeared normal but there were a few small areas of cystic degeneration. The cells of the tumour were astrocytes, some protoplasmic and some fibrillary in varying proportion. Both types of astrocytes produce glial fibrils which pass through the cytoplasm of the cells. This tumour was an astrocytoma. Let us pass on to the leukaemia. The appearance of the gums was quite striking (Plate V). There was haemorrhage and small areas of ulceration on the lower lips and the gums were haemorrhagic and hypertrophied. Histological section showed a very heavy infiltration with leukaemic cells. Petechial haemorrhages were present beneath all the serous membranes, particularly in the epicardium and also in the myocardium but the heart was otherwise normal. The right ovary showed a haemorrhagic cyst, no luteal cells were found histologically and there was no clear evidence that ovulation had occurred. The lungs were oedematous and there was a little fibrin on the pleural surfaces. Distinct areas of haemorrhage and leukaemic infiltration were present. Neither the liver nor the spleen were enlarged. Section of the liver showed a mild degree of diffuse leukaemic infiltration both in the sinusoids and the portal areas. The lymph nodes were enlarged from heavy leukaemic infiltration of the sinusoids and medulla with compression of the follicles. There was haemorrhage into the lymph-nodes and erythrophagocytosis. The sinusoids contained occasional giant cells. The blood film showed no mature granulocytes at all. There were, however, a very large number of nucleated cells and nearly all of them were atypical "blast" cells with irregular lobulated nuclei. The red marrow had extended all the way up the shaft of the femur. A section showed little except a heavy diffuse infiltration throughout with leukaemic cells. To sum up, this was a girl of ten who had a solid astrocytoma of the cerebrum two years ago for which a successful by-pass operation was performed. She received some radiotherapy and died suddenly from acute leukaemia with leukaemic infiltration in the marrow, the lungs, the lymph nodes and the gums. The terminal events were haemorrhage into the thalamus and acute pulmonary oedema.

*Prof. Neale:* Do we happen to know the relation of the tumour to the hypothalamus? I ask because of the possibility of neurogenic precocities.

*Dr. Johnson:* The hypothalamic nucleus on the left side was certainly compressed. It is of interest that when she first attended with her tumour she was described as a lean girl; when she presented subsequently she was obese. I had wondered whether this was a precocious puberty but I was unable to prove it.

*Dr. Cates:* Dorothy Russell has recently described the very marked changes in the blood vessels of the brain when there has been radiotherapy to a tumour and considers that a lot of destruction can be caused in normal tissue while the tumour is being bombarded. Are you in a position to make any observation on that point here?

*Dr. Johnson:* I do not think I am. These blood vessels that I have in my sections appear to be the normal blood vessels encountered in normal brain left behind when the astrocytoma has infiltrated round about.

*Dr. Cates:* Do you think the bleeding in that site might have been determined by radiotherapy?

*Dr. Johnson:* There was no evidence of it.

*Prof. Hewer:* Was the haemorrhage not the haemorrhage of leukaemia?

*Dr. Johnson:* I am sure that it was. The area of haemorrhage was quite apart from the tumour.

*Prof. Hewer:* One question that comes into our minds is that the leukaemia may have had some relation to the radiotherapy.

*Dr. Tudway:* First of all dealing with the more general question of radiotherapy

That was undertaken in this case in the absence of detailed knowledge of what was wrong with the patient except the assumption that there was a glioma. We don't usually like to treat tumours which we know are well-differentiated astrocytomas, which bears on Dr. Cates's question about damage to the brain because it is known that a certain dose will produce damage to blood vessels of the brain and the dose necessary to have a reliable effect on a well-differentiated astrocytoma would be very much the same dose—so there would be no question of trying to destroy the tumour directly. The only effect one might have by analogy with other tumours is to cause the cells to differentiate to a degree where they can no longer reproduce but I think that is a bit theoretical. Had we known this was as differentiated as it was we would not have taken the case on at that stage. However, it was treated on the basis that it might be a more undifferentiated tumour and therefore a wider area was treated than appeared to be necessary from the post mortem and to a fairly low dose, in fact about 3,000 r. This is not a dose which we would expect to have a damaging effect on the normal tissues.

As far as the question of a link between the occurrence of a leukaemia and the radiation goes, there has been a recent publication which appears to demonstrate that if cases of acute leukaemia in children are examined the mothers will have been examined in pregnancy with diagnostic X-ray to the pelvis twice as frequently as in a control group which nearly, but not quite I think, means that acute leukaemia in childhood is twice as likely if the mother has had diagnostic X-ray of the pelvis during pregnancy. Again Court-Browne, working for the M.R.C., has shown that irradiation of ankylosing spondylitis results in an increased leukaemia rate afterwards in these patients, usually adults. The likelihood of acute leukaemia appears to depend entirely on how much irradiation has been given. With the dosage most often used in adults the patients are about ten times as likely to get a leukaemia although of course this does not mean that they are very likely to get a leukaemia but their chances are definitely increased (from about 1 in 20,000 to 1 in 2,000). Americans collecting cases of carcinoma of the thymus found that quite a number had had irradiation of the thymus in infancy. In all these cases the increased risk of tumour formation arises in the part which actually received the irradiation. Let us try to bring this back to this particular case discussed here. If it were likely that it was irradiation of the bone marrow which would determine the onset of the leukaemia there really wasn't a high proportion of bone marrow irradiated. As both brain tumours and leukaemia are both not very uncommon as we see them I should have thought it was most likely in this instance that the leukaemia was fortuitous. This is, of course, only an expression of what I think is the probability and it is impossible to say definitely.

*Prof. Perry:* Could Dr. Tudway say anything about the true relationship in this case? Experimentally there appears to be a definite relationship between the dose and the period after which the leukaemia appears and I thought this applied in the leukaemias following spondylitis and pelvimetry. We are all very sensitive about this. I have a female patient who before valulotomy four years ago had a lot of X-rays because we wanted to know whether her mitral stenosis was better or not. She now has a myeloid leukaemia. Did we give it to her? This child had a two-year interval.

*Dr. Tudway:* I think this is rather a short interval but in Loutit's experimental cases he has conditions extremely carefully controlled. In the case of pelvimetry in the women conditions are relatively constant and much the same dose will be given in each case, for much the same reasons, and about the same time in the pregnancy. In this brain tumour only a part of the patient has been treated with a much higher dose. It is impossible to relate these different conditions to the expected time interval.

*Dr. Cates:* In the atom-bomb leukaemia is it not a fact that, although most of the cases came 7–10 years afterwards, some cases began to appear a year or so after the bomb dropped?

*Dr. Tudway:* That is so, but not very many straight away. The curve of the inciden[illegible] began to rise. We are dealing with probabilities.

*Prof. Perry:* Most of those near enough to the bomb to get a big dose died, howeve[illegible] The survivors who got leukaemia were protected if they were near or only got a reduc[illegible] dose.

*Dr. Tudway:* One suggestion is that in certain circumstances it may be the fai[illegible] small dose—for example to a leukoplakic area on the tongue—which will stimul[illegible] neoplasia, not the larger dose. I think there might be a critical dose which would j[illegible] do it.

Dr. Bolton then gave figures for the incidence of leukaemia in the years 1947[illegible] in Hiroshima atom-bomb survivors at varying distances from the centre of the atom[illegible] explosion and commented on this evidence of linear relationship of incidence to t[illegible] amount of radiation received:

| *Distance from centre (metres)* | *Approximate dosage received (r)* | *Incidence (per 10,000 survivors)* |
|---|---|---|
| 2,000 | 8 | 2 |
| 1,500–2,000 | 50 | 3–4 |
| 1,000–1,500 | 350 | 28 |
| 1,000 | More than 1,400 | 128 |

Dr. Tudway subsequently pointed out that a certain amount of shielding by buil[illegible] ings at all distances must have occurred thereby modifying the actual dose receiv[illegible] by partially protected individuals and that one cannot infer with certainty wheth[illegible] the relationship between dose and the incidence of leukaemia is a curvilinear or[illegible] linear one.

PLATE V

*Post-mortem appearance of the lips and gums shows hypertrophy due to leukaemic infiltration, haemorrhage and ulceration. There is a petechial haemorrhage on the right cheek.*

## Figures and Tables

**Figure f1:**
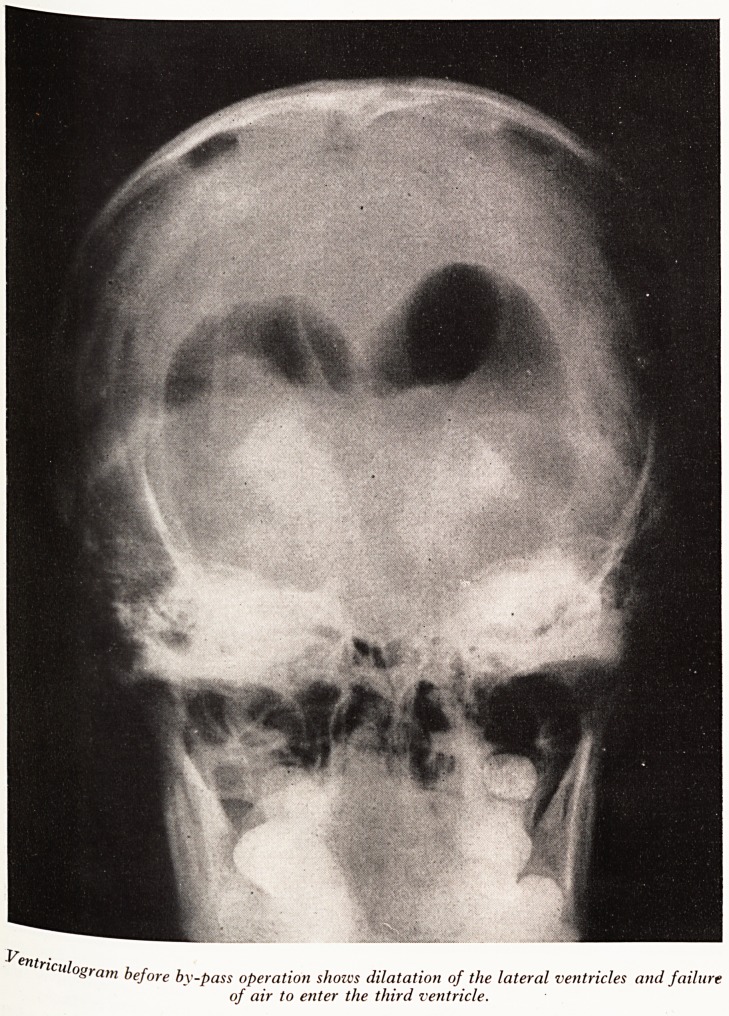


**Figure f2:**
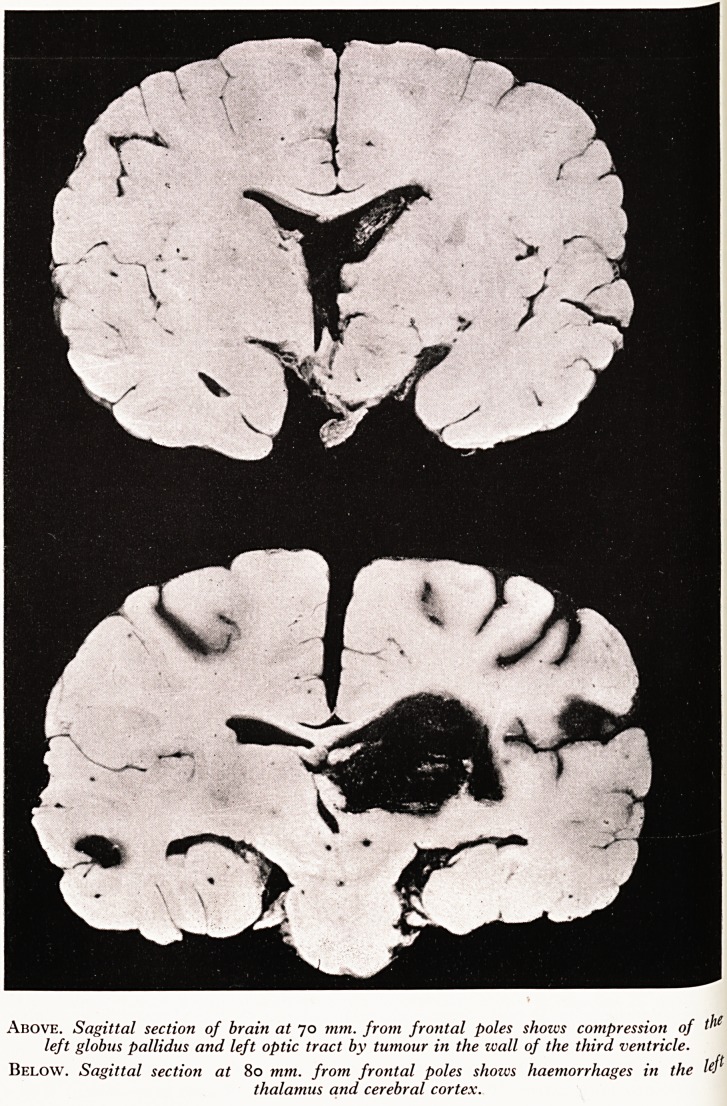


**Figure f3:**